# Supplementary material for: The negative regulator SMAX1 controls mycorrhizal symbiosis and strigolactone biosynthesis in rice
Source: Nat Commun. 2020 Apr 30;11:2114. doi: 10.1038/s41467-020-16021-1 (PMC7193599; doi:10.1038/s41467-020-16021-1)
Supplement: Supplementary file 1 — Supplementary Information [file 41467_2020_16021_MOESM1_ESM.pdf]

List of Supplementary Figures

Supplementary Figure 1. The phylogenetic tree of SMAX-like (SMXL)s family

Supplementary Figure 2. SMAX1 protein structure and sequences

Supplementary Figure 3. Subcellular localization of OsD14L, D3 and SMAX1

Supplementary Figure 4. Mutant analysis of *SMAX1* T-DNA insertion lines

Supplementary Figure 5. Characterisation of symbiosis phenotype of *smax1* alleles.

Supplementary Figure 6. AM marker gene expression analysis of *the smax1-1*^{SMAX1} complementation line

Supplementary Figure 7. Mutant analysis of *dwarf14-like (d14l)* CRISPR/Cas9 edited lines

Supplementary Figure 8. AM colonisation of *dwarf14-like (d14l)* CRISPR/Cas9 edited lines

Supplementary Figure 9. AM marker gene expression analysis of *dwarf14-like (d14l)* CRISPR/Cas9 edited lines

Supplementary Figure 10. AM marker gene expression analysis of *d14l/smax1* double knockout mutant

Supplementary Figure 11. AM marker gene expression analysis of *d3/smax1* double knockout mutant

Supplementary Figure 12. SMAX1-GFP protein level in wild-types, *d14l* and *d3* mutant protoplasts

Supplementary Figure 13. Mesocotyl length of *smax1, d3* and d3/*smax1* double knockout

Supplementary Figure 14. Validation of RNAseq data analysis by quantitative RT-PCR and gene induction in response to AM symbiosis development in the wild-type

Supplementary Figure 15. SMAX1-regulated gene expression analyses in *smax1*^{SMAX1} complementation lines Supplementary Figure 16. Gene expression analysis of SMAX1-regulated genes in *d14l*

Supplementary Figure 17. Strigolactone biosynthesis gene expression by quantitative RT-PCR

Supplementary Figure 18. Measurement of strigolactones (SLs), 4-deoxyorobanchol (4-DO) and methoxy-5-deoxystrigol isomer 1 (MeO-5-DS is1) in rice

Supplementary Figure 19. Gene ontology enrichment analysis of genes down-regulated by *smax1* mutation

List of supplementary Table

Supplementary Table 1. Mapping summary of the RNAseq reads

Supplementary Table 2. Functionally characterised *smax1*-UP genes in AM symbiosis

Supplementary Table 3. Primers used in this study

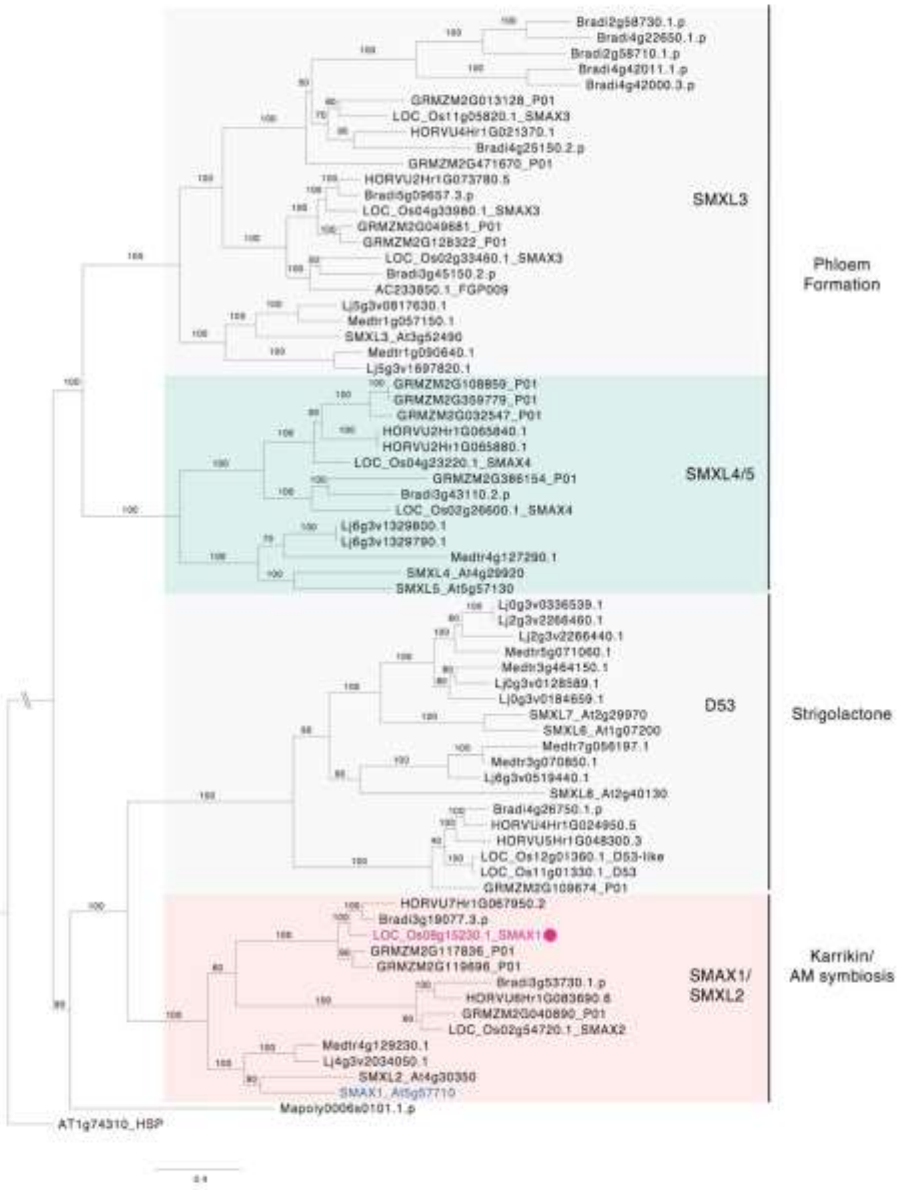

Supplementary Figure 1. The phylogeny tree of SMAX-like (SMXL)s family

The tree represents the phylogenetic relationship among SMXL sequences from Arabidopsis, rice, *Brachypodium distachyon*, maize, barley, *Medicago truncatula* and *Lotus japonicus*. Rice SMAX1 is marked with red circle. Arabidopsis Heat Shock Protein 101 was used as an outgroup.

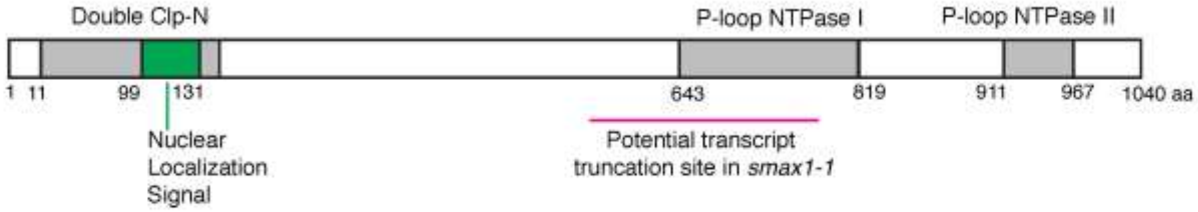

**1-11**		MRADLSTIQQ
**11-135**	**Double Clp-N**	TLTPEAAAALARAMDEAGRRRHGQTTPLHVAAALLAAPAGLLRQACARAASAAGVGGGGGAAAGAGAG<br>AHPLHCRALELCFSVALDRLPAAAAAAAAAHGAGASPPVSNALVAALKRAQAQQRRG
**135-643**		CPEAAQQPLLAVKVELEQLVLSILDDPSVSRVMREASFSSAAVKSIIEQSLSAPSPCPSAAASTTTAGPGP<br>LSPSPSPLPRAGAANAYLNPRLAAAAAVASGGGGGGGDDARKVIDVMLKPTRRNPVLVGDAGPDAVLKE<br>AIRRIPTAGFPALAGAKVLPLEAELAKLAGDKAAMAARIGDLGAVVERLLGEHGGVVLDLGDLKWLVDGP<br>AAAASEGGKAAVAEMGRLLRRFGRAGVWAVCTAACTTYLRCKVYHPGMEAEWDLHAVPIARGGAPIAA<br>AAAGSALRPGGSGILNSSMGMLSPALRPMPVTPTALRWPPPGSDQSPAAKPAMCLLCKGSYERELAKLE<br>AEQTDKPASRPEAAKPGLPHWLQLSNDQNKAKEQELKLKRSKDELERKWRETCARIHSACPMAPALSVP<br>LATFTP*RPPVEPKLGVARGAAVPTLKMNPSWEKPSVAPTLELRKSPPASPVKTDLVLCRLDPGTNPAVEN<br>EQKESCEGLTALQKAKIAGISD
**643-819**	**NTPase I**	IESFKRLLKGLTEKVSWQSDAASAIAAVVIQCRSGSGKRRNVGTRGDMWLLFVGPDQAGKRKMVNALSE<br>LMANTRPVVVNFGGDSRLGRVGNDGPNMGFWGKTALDRVTEAVRQNPFSVIVLEGIDQVDVVVHGKIK<br>RAMETGRLPDSRGREVSLG*NVIFVLTTNWVPEELKGSNV
**819-911**		ETLLRGEERMLESTSSSWQLELSIGDKQVKHRADWLCDDVRPAKLAKELSSSHGLSLDLNLAVGALDDT<br>EGSHNSSDVSVEQEQEKGQLAV
**819-967**	**NTPase II**	KRSTPAPGSDILELVDDAIVFRPVDFTPFRKTVTDCISAKFESVMGSSSSFRIDEDA
**968-1040**		VDWMVGSVWLTDEKIEDWAEKVLKPSIERLWHNVKHDSGRSIIRLTAVAAKALPRWGGGREGLPVAVTIA<br>IDGM

Supplementary Figure 2. SMAX1 protein structure and sequences

The diagram represents the SMAX1 protein structure with known domains indicated as grey boxes and their corresponding protein sequences. The potential nuclear localisation signal is highlighted in green and the potential transcript truncation site of *smax1-1* is marked between asterisks in red.

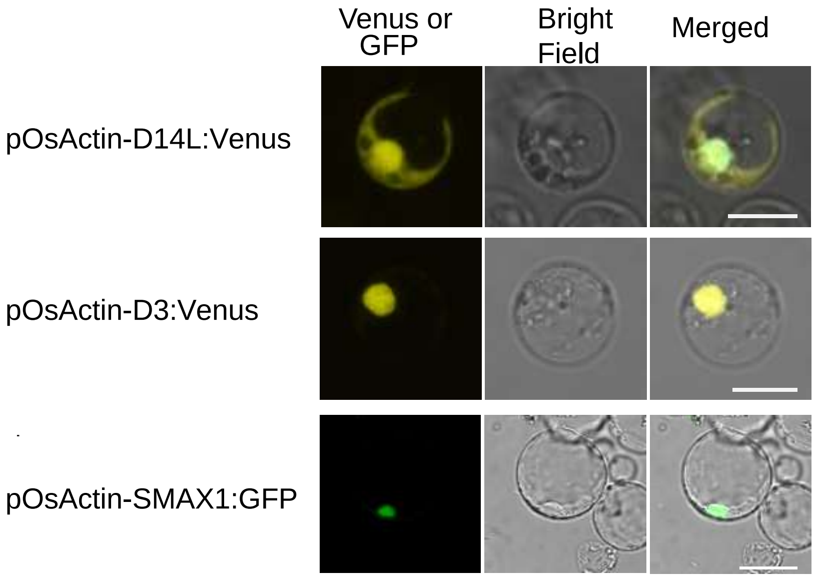

Supplementary Figure 3. Subcellular localisation of OsD14L, D3 and SMAX1

Subcellular localisation of D14L, D3 and SMAX1 in rice leaf protoplast was visualised by confocal microscopy. Coding regions of each gene were fused with a fluorescent protein, either Venus or GFP as indicated, and transiently expressed under the control of the promoter of the rice *Actin1* housekeeping gene. Three protoplasts per construct were microscopically analysed. scale = 25 μm.

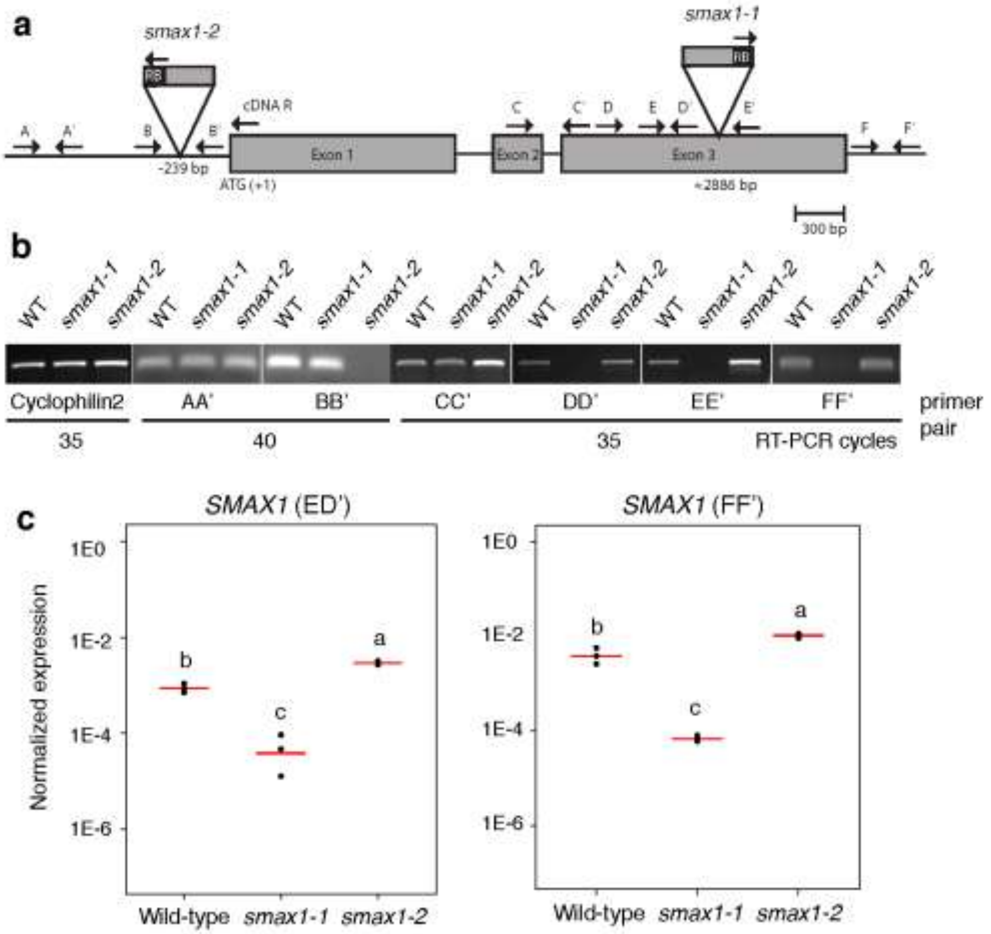

Supplementary Figure 4. Mutant analysis of *SMAX1* T-DNA insertion lines

a. The diagram indicates the sites of the T-DNA insertions and primers used for reverse transcription (RT) **(b)** and quantitative RT-PCR **(c);** RB: right border.

b. RT-PCR amplicons indicate the presence and absence of *SMAX1* transcript in two *smax1* mutants compared to wild-type.

c. Quantitative RT-PCR quantified the expression levels of *SMAX1* shortly before and after T-DNA insertion sites. Relative gene expression values were normalised to the geometric mean of the three housekeeping genes, *Cyclophillin2, Ubiquitin and Glyceraldehyde 3-phosphate dehydrogenase (GAPDH),* and shown relative to *Cyclophilin2*. Each dot and red bar indicate individual plant and mean values of three replicates. Kruskal-Wallis test ($p<0.05$) was used, and followed by the *post hoc* tests according to the Agricolae package in the R software. Different letters represent significant difference (p-value <0.05). Degrees of freedom =2, n=3 per genotype, *SMAX1* (ED'): χ^2=7.2, p=0.03; *SMAX1* (FF'): χ^2=7.2, p=0.03.

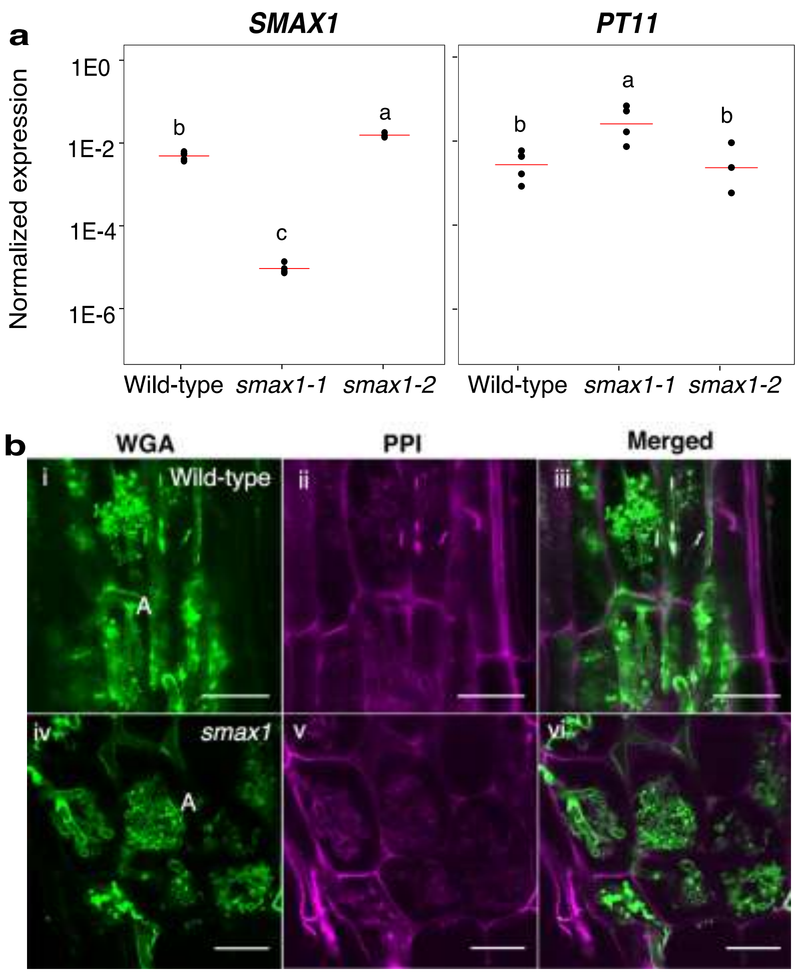

Supplementary Figure 5. Characterisation of symbiosis phenotype of *smax1* alleles

a. Quantitative RT-PCR shows the expression level of *SMAX1* and the AM marker *Phosphate transporter 11 (PT11)*, in colonised wild-type and the two *smax1* mutant alleles. Kruskal-Wallis test ($p<0.05$) was used, and followed by the *post hoc* tests according to the Agricolae package in the R software. Different letters represent significant difference (p-value <0.05). Each dot and red bar indicate values from an individual plant and the mean value of each genotype. Degrees of freedom =2, wild-type (n=5), *smax1-1* (n=4), *smax1-2* (n=4), *SMAX1*: χ^2=9.69, p=0.008; *PT11*: χ^2=6.49, p=0.04.

b. Arbuscule (A) structures were visualised by observing colonised roots stained with Wheat Germ Agglutinin (WGA, green) fluorescent dye conjugated with Alexa-488 by confocal microscopy (i,iv). Propidium iodide (PPI, magenta) staining of the same roots for plant cell walls (ii, v) were merged (iii, vi). Images represent arbuscule structures from at least three independent plants of each genotype. Scale bar 10 μm.

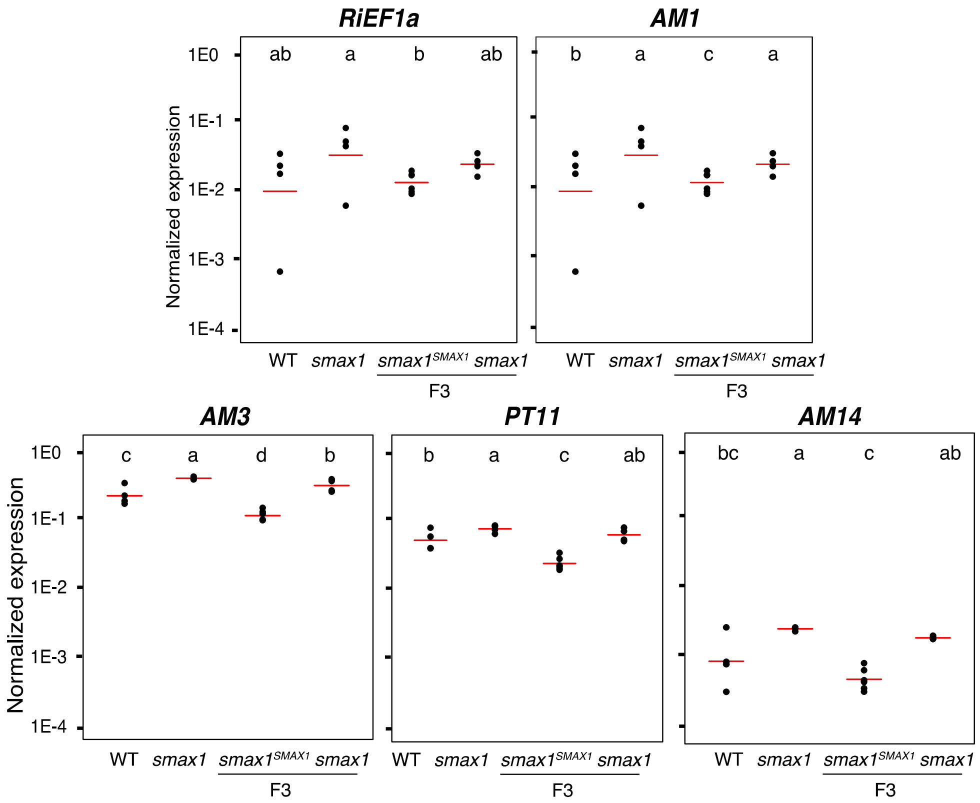

Supplementary Figure 6. AM marker gene expression analysis of *the smax1-1^{SMAX1}* complementation line.

Quantitative RT-PCR based analysis of the AM marker genes and the *R. irregularis* housekeeping gene *Elongation Factor1a* (*RiEF1a*) in roots of *smax1-1* mutant and the *smax1-1^{SMAX1}* complemented line inoculated with R. irregularis. Kruskal-Wallis test ($p<0.05$) was used, followed by the *post hoc* tests according to the Agricolae package of the R software. Different letters represent significant difference (p-value <0.05). Each dot and red bar indicate values from an individual plant and the mean value of each genotype. Degrees of freedom =3, wild-type (n= 4), *smax1* (n=4), *smax1-1^{SMAX1}* (F3, n= 6), *smax1-1* (F3, n=4), *RiEF1a*: χ^2=4.78, p=0.19; *AM1*: χ^2=12.50, p=0.006; *AM3*: χ^2=15.07, p=0.002; *PT11*: χ^2=13.18, p=0.004; *AM14*: χ^2=10.61, p=0.014.

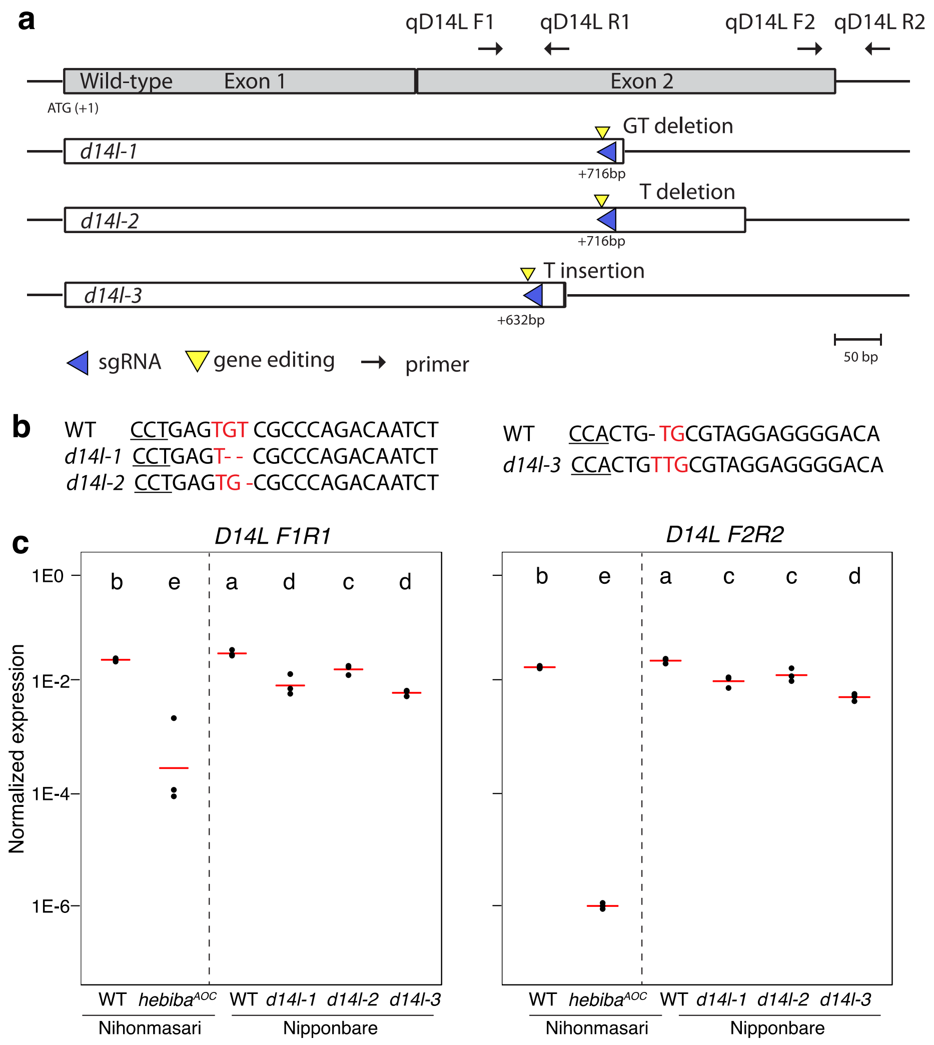

Supplementary Figure 7. Mutant analysis of *dwarf14-like (d14l)* CRISPR/Cas9 edited lines

a. The diagram shows the site of sgRNA complementarity, gene editing event and primer sets used for *D14L* gene expression analysis. **b.** Detail of *d14l* CRISPR/Cas9 edited sequences. **c**. Quantitative RT-PCR based analysis of the expression levels of *D14L* before and after CRISPR/Cas9 editing. Kruskal-Wallis test ($p<0.05$) was used, followed by the *post hoc* tests according to the Agricolae package of the R software. Different letters represent significant difference (p-value <0.05). Each dot and red bar indicate values from an individual plant and the mean value of each genotype (n=3). Degrees of freedom =5, n=3 for each genotype, *D14L* (F1R1): $\chi^2=16.02$, p=0.007; *D14L* (F2R2): $\chi^2=16.02$, p=0.007.

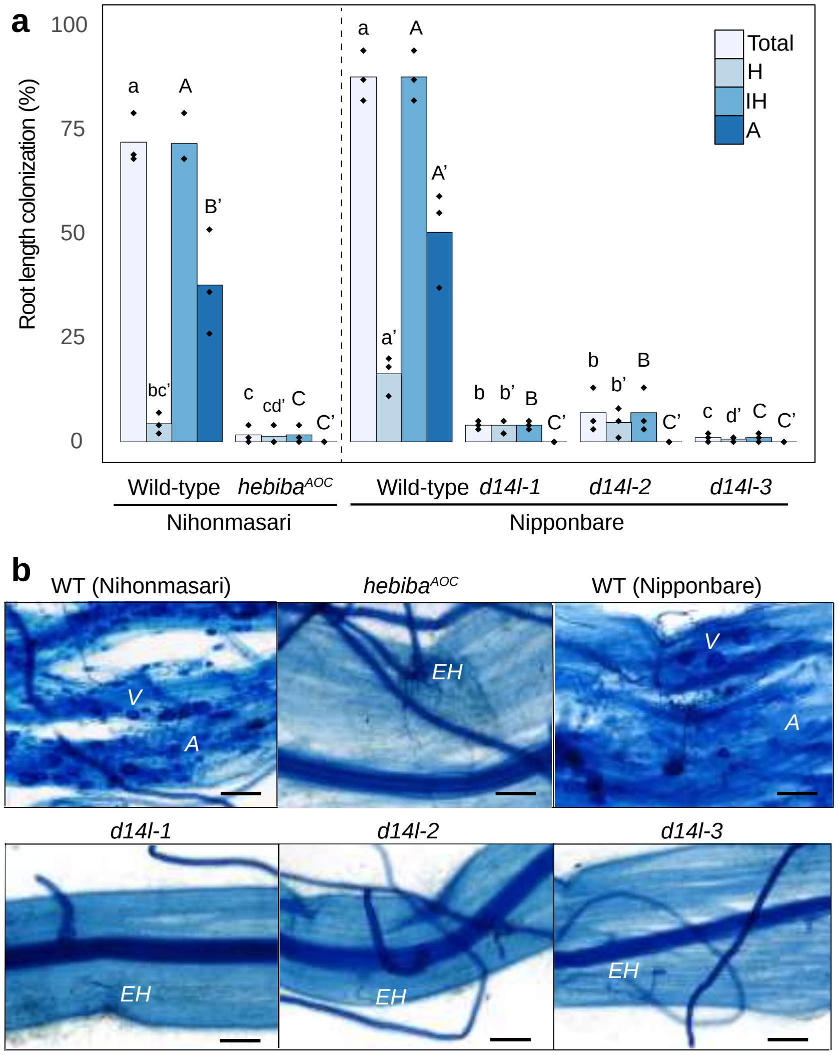

Supplementary Figure 8. AM colonisation of *dwarf14-like (d14l)* CRISPR/Cas9 edited lines

a. AM colonization levels of previously published *d14l* mutant (*hebiba AOC*) was compared with new *d14l* CRISPR lines at 7 wpi with 300 spores. Each bar indicates the average percentage value of the respective fungal structure. Total, total colonization, H, hyphopodia, IH, intraradical hyphae, A, arbuscules. Kruskal-Wallis test ($p<0.05$) was used, followed by the *post hoc* tests according to the Agricolae package of the R software. Different letters represent significant difference (p-value <0.05). Degrees of freedom = 5, n=3 per genotype, Total: $\chi^2=14.87$, $p=0.01$; H: $\chi^2=12.24$, $p=0.03$; IH: $\chi^2=14.89$, $p=0.01$; A: $\chi^2=16.54$, $p=0.005$.

b. Micrographs of trypan blue stained roots for fungal structures. V, vesicles, A, arbuscule, EH, extraradical hyphae; scale 200 μm. Images represent at least three independent plants per genotype.

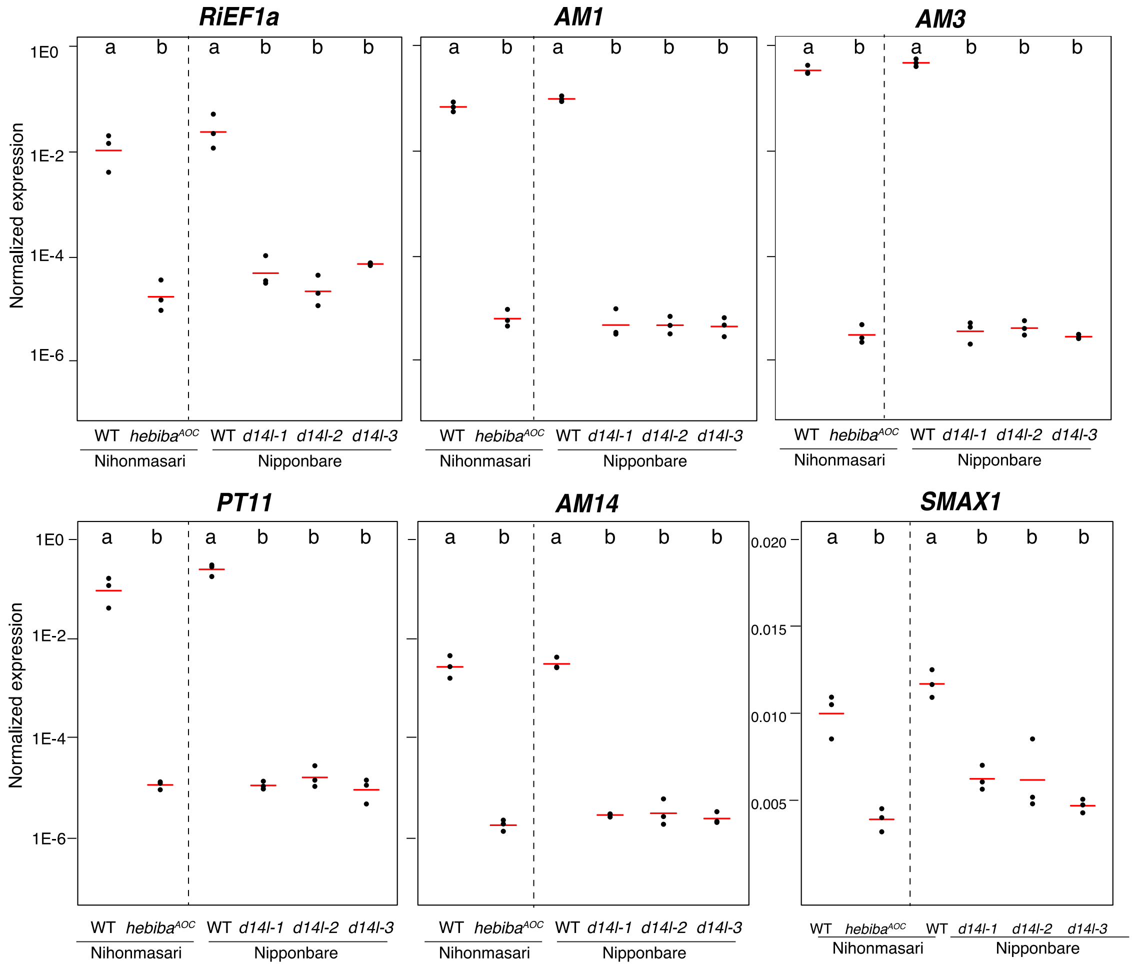

Supplementary Figure 9. AM marker gene expression analysis of *dwarf14-like (d14l)* CRISPR/Cas9 edited lines

Quantitative RT-PCR based analysis of the expression levels of *R. irregularis* housekeeping gene *Elongation Factor1a* (*RiEF1a*), AM marker genes (*AM1, AM3, PT11 and AM14*) and *SMAX1* in roots inoculated with *R. irregularis* (compare Supplementary Figure 8). Each dot and red bar indicate values from an individual plant and the mean value of each genotype. Relative gene expression values were normalised against the geometric mean of the rice housekeeping genes, *Cyclophillin2, Ubiquitin* and *GAPDH,* and are shown relative to *Cyclophillin2*. One-way ANOVA (p<0.05) was used, followed by the *post hoc* tests according to the Agricolae package of the R software. Different letters represent significant difference (p-value <0.05), n=3 per genotype, F $(RiEF1a)_{5,12}$ =71.35; F $(AM1)_{5, 12}$ = 499.39; F$(AM3)_{5,12}$=1134.7; F$(PT11)_{5,12}$=347.26; F$(AM14)_{5,12}$=280.26; F$(SMAX1)_{5,12}$=22.34, p<00001).

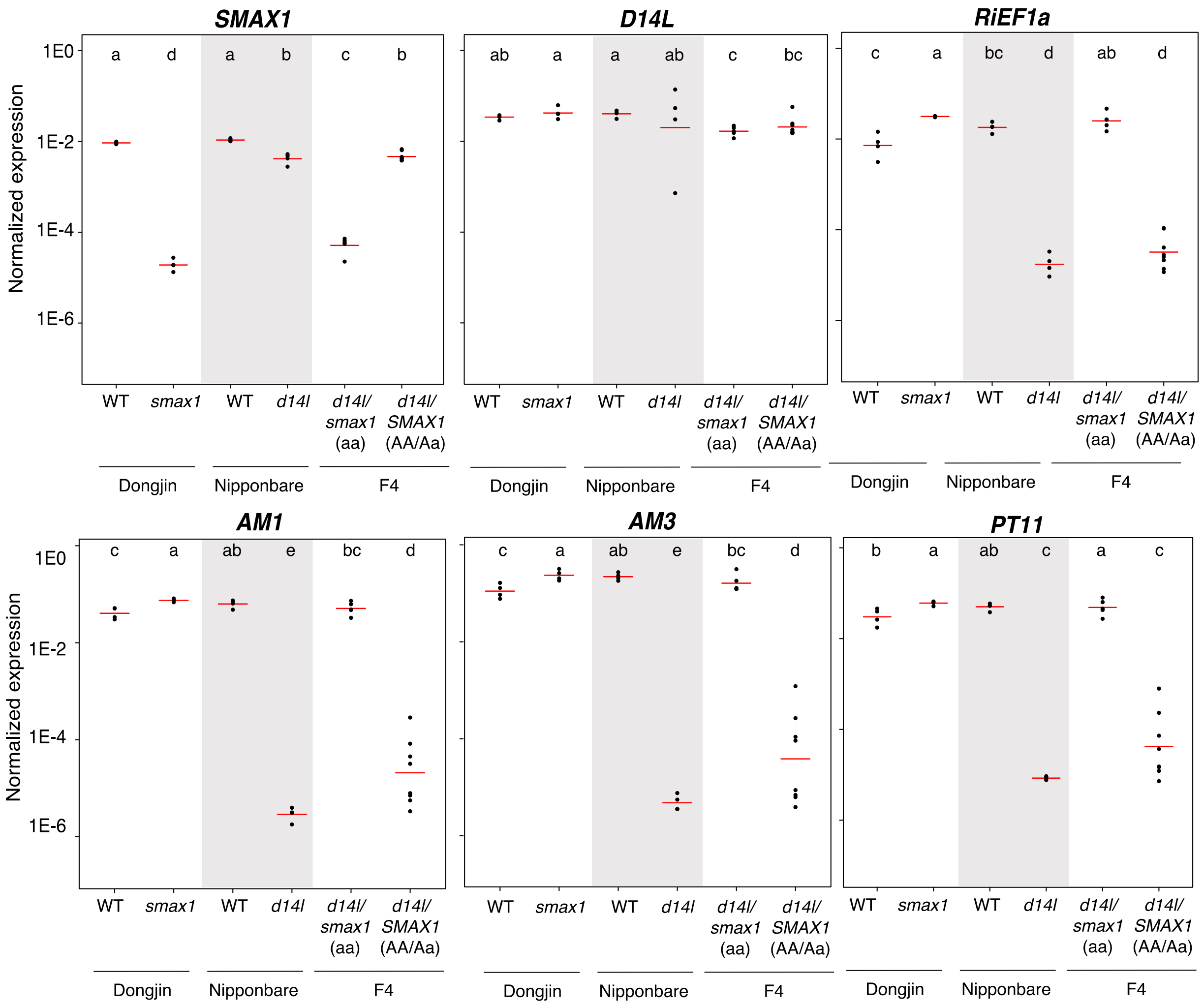

Supplementary Figure 10. AM marker gene expression analysis of *d14l/smax1* double knockout mutant

Quantitative RT-PCR based analysis of the expression levels of *R. irregularis* housekeeping gene *Elongation Factor1a* (*RiEF1a*), AM marker genes (*AM1, AM3, PT11 and AM14*), *SMAX1* and *D14L* in roots inoculated with *R. irregularis* (compare Figure 2). Each dot and red bar indicate values from an individual plant and the mean value of each genotype. Relative gene expression values were normalised against the geometric mean of the rice housekeeping genes, *Cyclophillin2, Ubiquitin* and *GAPDH,* and are shown relative to *Cyclophillin2*. Kruskal-Wallis test (p<0.05) was used, followed by the *post hoc* tests according to the Agricolae package of the R software. Different letters represent significant difference (p-value <0.05). Degrees of freedom =5, Dongjin wild-type (n=4), *smax1*(n=4), Nipponbare wild-type (n=4), *d14l*

(n=4), *d14l/smax1* (n=5), *d14l/SMAX1* (n=8), *SMAX1*: χ^2=25.56, p=0.0001; *D14L*: χ^2=13.07, p=0.02; *RiEF1a*: χ^2=24.92, p=0.0001; *AM1*: χ^2=24.8, p=0.0002; *AM3*: χ^2=24.18, p=0.0002; *PT11*: χ^2=23.54, p=0.0003.

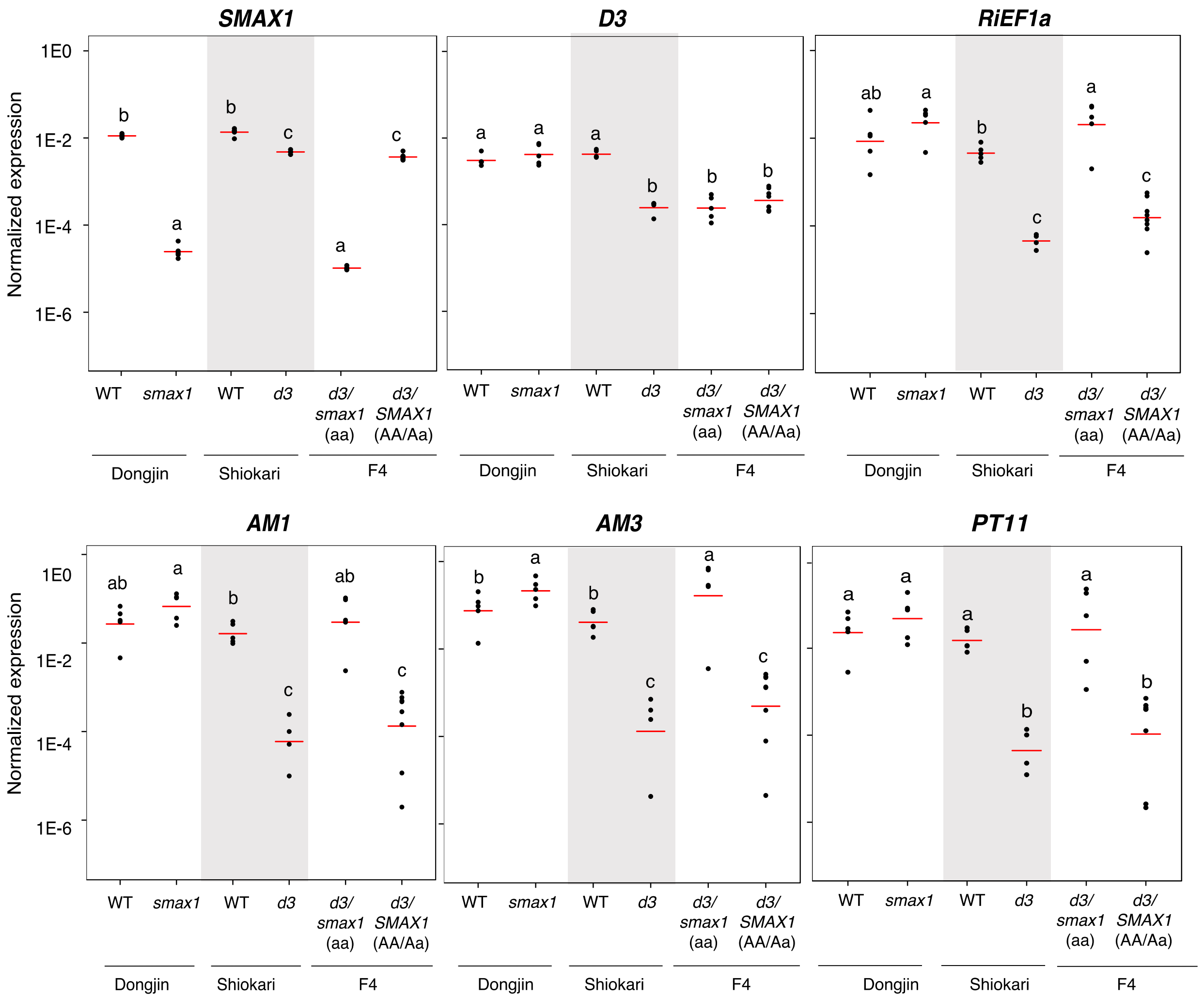

Supplementary Figure 11. AM marker gene expression analysis of *d3/smax1* double knockout mutant

Quantitative RT-PCR based analysis of the expression levels of *R. irregularis* housekeeping gene *Elongation Factor1a* (*RiEF1a*), AM marker genes (*AM1, AM3, PT11 and AM14*), *SMAX1* and *D3* in roots inoculated with *R. irregularis* (compare Figure 2). Each dot and red bar indicate values from an individual plant and the mean value of each genotype. Relative gene expression values were normalised against the geometric mean of the rice housekeeping genes, *Cyclophillin2, Ubiquitin* and *GAPDH,* and are shown relative to *Cyclophillin2*. Kruskal-Wallis test ($p<0.05$) was used, followed by the *post hoc* tests according to the Agricolae package of the R software. Different letters represent significant difference (p-value <0.05). Degrees of freedom =5, Dongjin wild-type (n=5), *smax1* (n=5), Shiokari wild-type (n=5), *d3* (n=4), *d3/smax1* (n=5), *d3*/SMAX1 (n=8), *SMAX1*: χ^2=26.01, p=0.0001; *D3*: χ^2=24.09, p=0.0002; *RiEF1a*: χ^2=24.81, p=0.0002; *AM1*: χ^2=24.61, p=0.0002; *AM3*: χ^2=25.49, p=0.0001; *PT11*: χ^2=23.1, p=0.0003.

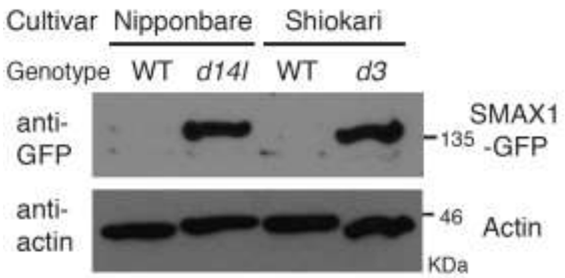

Supplementary Figure 12. SMAX1-GFP protein level in *d14l* and *d3* protoplasts

Western blot showing protein levels of SMAX1-GFP in leaf protoplasts of *d14* and *d3* mutant and corresponding wild-type cultivars Nipponbare and Shiokari, respectively. Actin served as a loading control and was detected by an anti-Actin antibody.

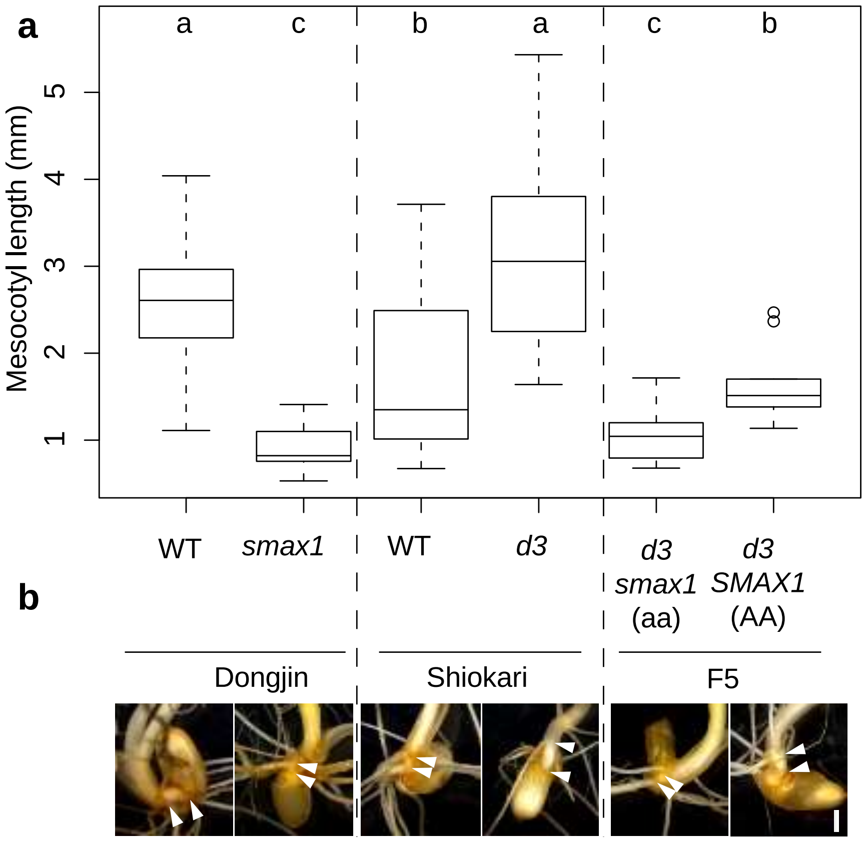

Supplementary Figure 13. Mesocotyl length of *smax1, d3* and d3/*smax1* double knockout

a. Mesocotyl length of 7-day old seedlings. The center line of the box plot indicates median; box limits are upper and lower quartiles; whiskers are 1.5x interquartile range; open circles represent outliers. Kruskal-Wallis test (p<0.05) was used followed by the *post hoc* tests according to the Agricolae package in R software. Different letters represent significant difference (p-value <0.05), degree of freedom =5, Dongjin wild-type (n=13), *smax1 (*n=10), Shiokari wild-type (n=14), *d3 (*n=9), *d3/smax1 (*n=10), *d3*/*SMAX1* (n=10), χ^2=38.25, p=3.4E-7. **b.** Representative images of the mesocotyl for each genotype as indicated. WT, wild-type, Dongjin and Shiokari are the corresponding cultivars of the respective mutants. F5 indicates the 5th generation progeny from the initial crossing. Scale bar 4 mm. Images represent mesocotyls of all plants analysed per genotype.

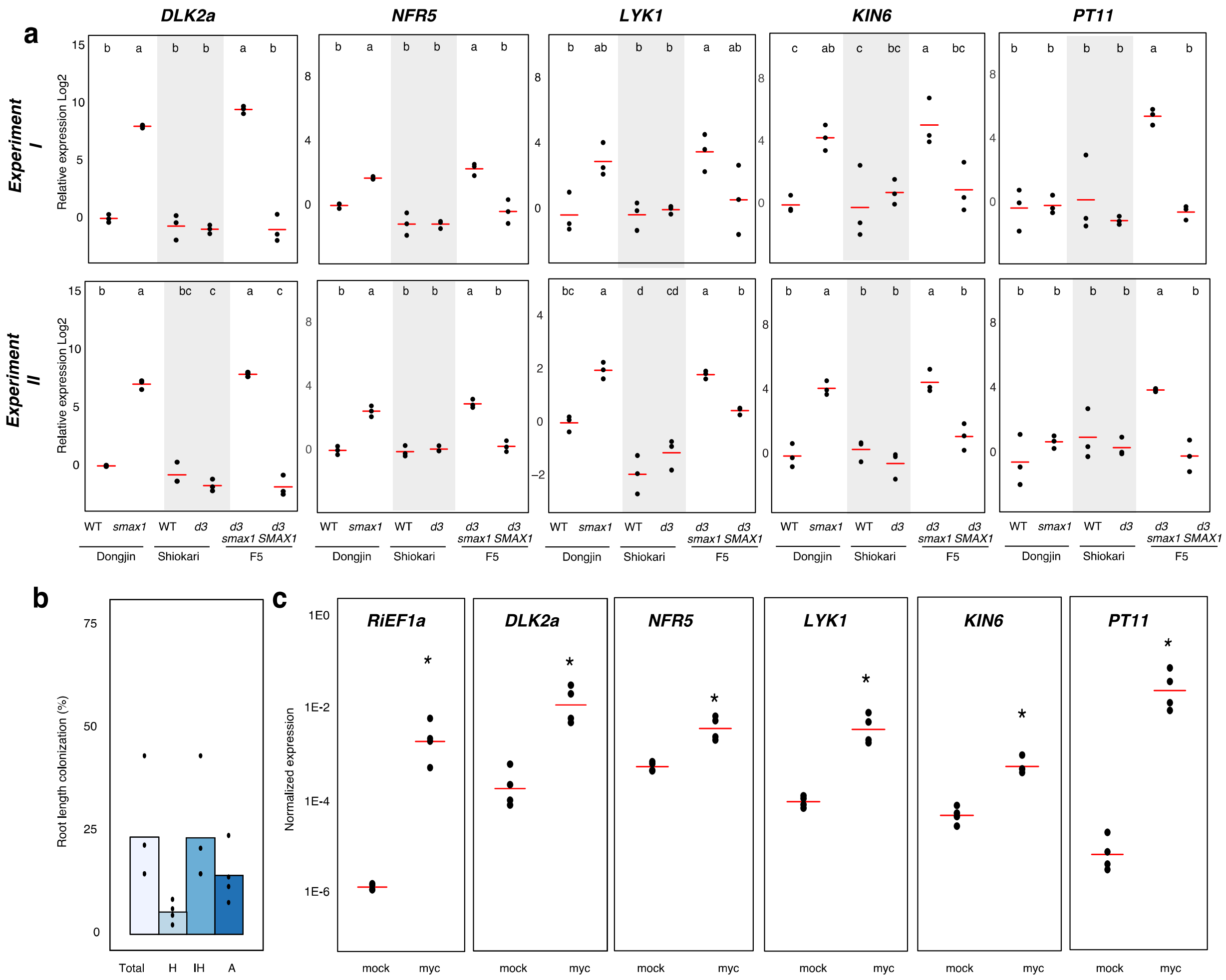

Supplementary Figure 14. Validation of RNAseq data analysis by quantitative RT- PCR and gene induction in response to AM symbiosis development in the wild-type

a. Analysis of the expression levels representative differentially expressed genes corresponding to the karrikin marker *DLK2a*, the LysM RLKs *NFR5* and *LYK1*, the kinase *KIN6* and the symbiosis phosphate transporter *PT11*. The qRT-PCR experiments were performed with cDNAs made from the RNA used for RNAseq sample library production (experiment I) and another independent experiment (experiment II). Each dot and red bar indicate values from an individual plant and the mean value of each genotype (n=3). Relative gene expression values were normalised against the geometric mean of the rice housekeeping genes*, Cyclophillin2, Ubiquitin* and *GAPDH,* and are shown as fold-change (log2 scale) compared to wild-type (Dongjin). One-way ANOVA (p<0.05) was used followed by the *post hoc* Tukey tests according to the Agricolae package of the R software. Different letters represent significant

difference (p-value <0.05). For Experiment I, n=3 per genotype, $F(\textit{DLK2})_{5,12}$ =143.46; $F(\textit{NFR5})_{5, 12}$ = 30.07; $F(\textit{LYK1})_{5,12}$=5.84; $F(\textit{KIN6})_{5,12}$=7.79; $F(\textit{PT11})_{5,12}$=12.3, p<0.01). For Experiment II, n=3 per genotype, $F(\textit{DLK2})_{5,12}$ =165.62; $F(\textit{NFR5})_{5, 12}$ = 64.19; $F(\textit{LYK1})_{5,12}$=40.66; $F(\textit{KIN6})_{5,12}$=28.33; $F(\textit{PT11})_{5,12}$=7.16, p<0.003). **b** and **c.** Microscopic observation (b) and qRT-PCR based expression analysis of marker genes showing significant gene induction in wild-type cultivar Dongjin colonised by *R.irregularis* (c). Total, total colonization, H, hyphopodia, IH, intraradical hyphae and A, arbuscule. Expression levels of the *R. irregularis* housekeeping gene, *Elongation Factor 1a* (*RiEF1a*), AM marker genes, *PT11* and four genes that were induced in *smax1* determined by qRT-PCR. Mock, non-inoculated control; myc, colonised by *R. irregularis*. Each dot and red bar indicate values from an individual plant and the mean value of each genotype (n=4). Mann-Whitney U-test (p<0.05) was used and asterisks above each bar represent statistically different groups compared to mock **(c)**.

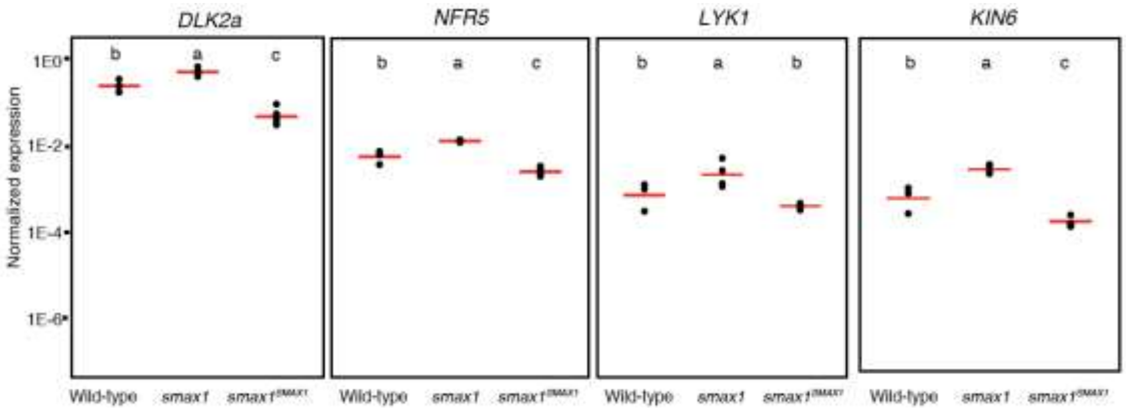

Supplementary Figure 15. *SMAX1*-regulated gene expression analyses in the *smax1*^{SMAX1} complementation line.

Four genes that were induced in *smax1* are quantified by qRT-PCR. Wild-type (Dongjin), *smax1* mutant and the complementation line (*smax1*^{SMAX1}). Each dot and red bar indicate values from an individual plant and the mean value of each genotype (n=4-8). Relative gene expression values were normalised against the geometric mean of the rice housekeeping genes, *Cyclophillin2, Ubiquitin* and *GAPDH,* and are shown relative to *Cyclophillin2*. Kruskal-Wallis test ($p<0.05$) was used, followed by the *post hoc* tests according to the Agricolae package of the R software. Different letters represent significant difference (p-value <0.05). Degrees of freedom =2, wild-type (n= 3), *smax1* (n=4), *smax1-1*^{SMAX1} (n= 5), *DLK2a*: χ^2=9.69, p=0.008; *NFR5*: χ^2=9.69, p=0.008; *LYK1*: χ^2=6.89, p=0.032; *KIN6*: χ^2=9.69, p=0.008.

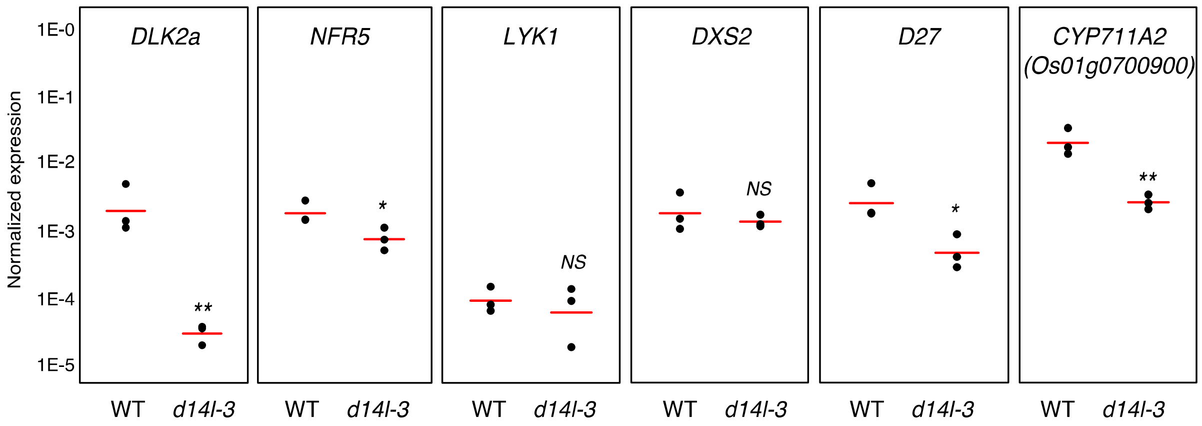

Supplementary Figure 16. Gene expression analysis of SMAX1-regulated genes in *d14l*

Analysis of the expression levels six SMAX1-regulated genes in *d14l* including three strigolactone biosynthesis genes *DXS2, D27* and *CYP11A2 (Os01g0700900)*. The qRT-PCR experiment was performed with cDNA from mock-inoculated control roots. Each dot and red bar indicate values from an individual plant and the mean value of each genotype (n=3). Relative gene expression values were normalised against the geometric mean of the rice housekeeping genes*, Cyclophillin2, Ubiquitin* and *GAPDH,* and is shown relative to *Cyclophillin2*. Two-tailed student t-test (p<0.05) was used according to the Agricolae package of the R software. Asterisk represents significant difference (p-value *<0.05, **<0.01, NS, not significant).

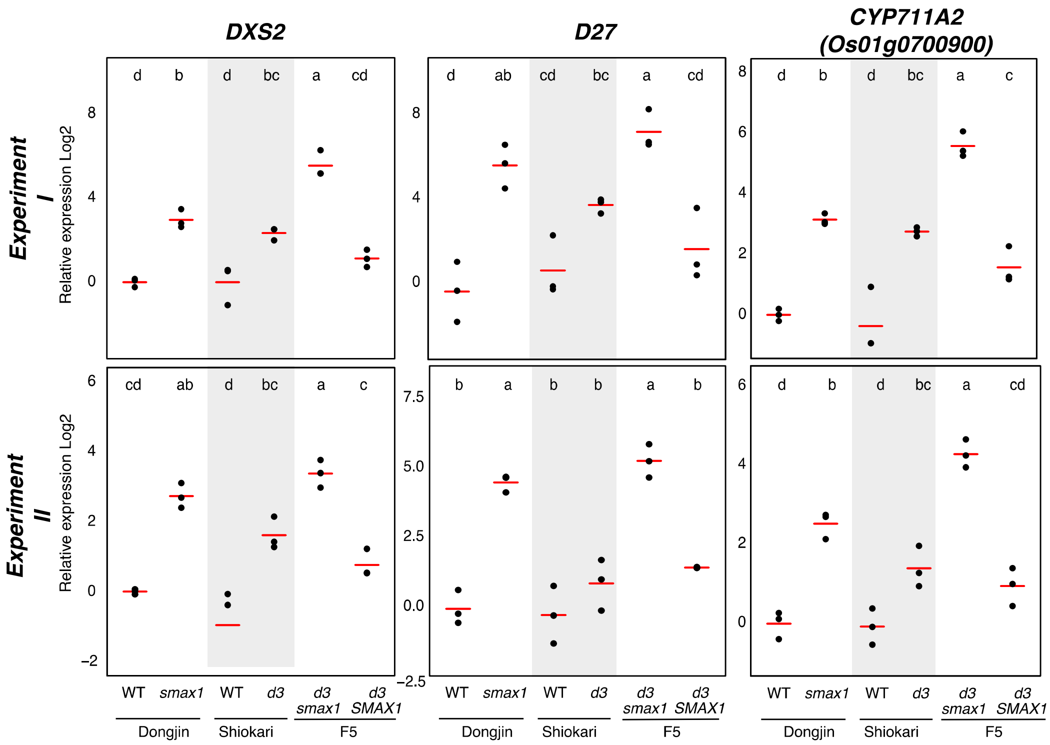

Supplementary Figure 17. Strigolactone biosynthesis gene expression by quantitative RT-PCR

Analysis of the expression levels three strigolactone biosynthesis genes *DXS2, D27* and *CYP711A2 (Os01g0700900)*. The qRT-PCR experiment was performed with cDNA from RNA of mock-inoculated control roots from two independent experiments, from the same RNA used for RNAseq sample library production (experiment I) and from a second experiment (experiment II). Each dot and red bar indicate values from an individual plant and the mean value of each genotype. Relative gene expression values were normalised against the geometric mean of the rice housekeeping genes*, Cyclophillin2, Ubiquitin* and *GAPDH,* and are shown as fold-change (log2 scale) relative to wild-type (Dongjin cultivar) . One-way ANOVA ($p<0.05$) was used followed by the *post hoc* Tukey tests according to the Agricolae package of the R software. Different letters represent significant difference (p-value <0.05). For Experiment I, n=3 per genotype, F(*DXS2*)$_{5,12}$ =44.22; F(*D27*) $_{5, 12}$ = 17.28; F(*CYP711A2*)$_{5,12}$=45.96, p<4.1E-05). For Experiment II, n=3 per genotype, F(*DXS2*)$_{5,12}$ =21.24; F(*D27*) $_{5, 12}$ = 36.59; F(*CYP711A2*)$_{5,12}$=46.8, p<1.4E-05).

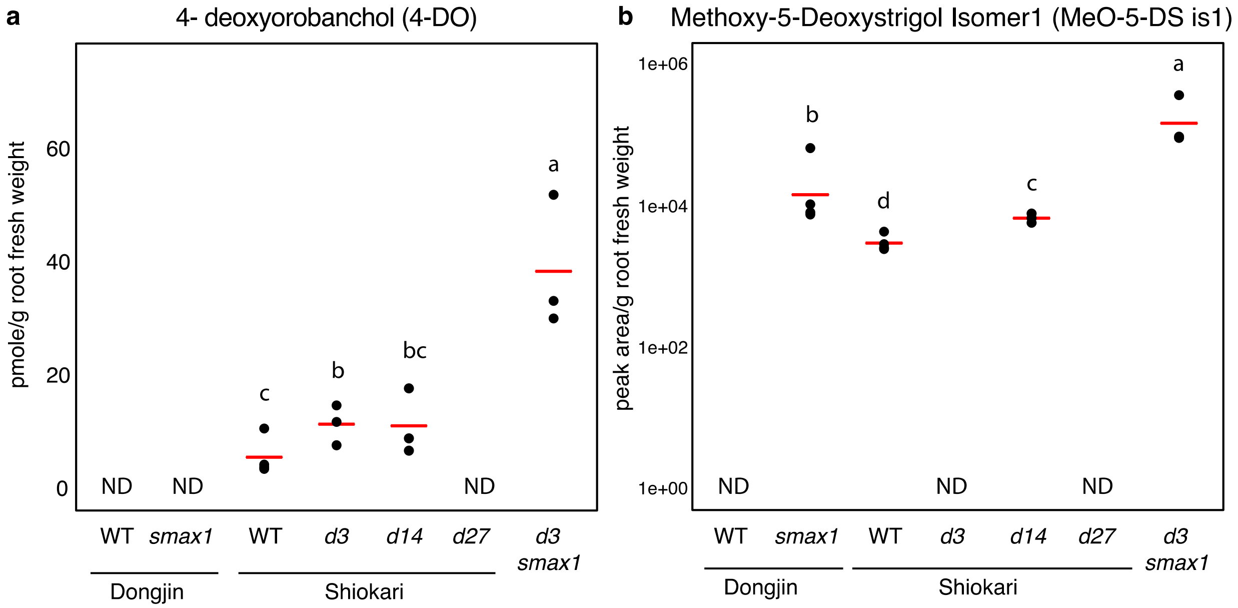

Supplementary Figure 18. Measurement of strigolactones (SLs), 4-deoxyorobanchol (4-DO) and methoxy-5-deoxystrigol isomer 1 (MeO-5-DS is1) in rice

The level of two SLs, 4-DO (a) and MeO-S-DS is1 (b), were measured in root exudates of 4-week old rice grown under low phosphate (40 uM) conditions. Each dot represents one biological replicate consisting of 2-5 plants. For statistical analysis, Kruskal-Wallis test ($p<0.05$) was used, followed by the *post hoc* tests according to the Agricolae package of the R software. Different letters represent significant difference (p-value <0.05). ND not-detected. For 4-DO, degree of freedom=3, n=3-4 per genotype, $\chi^2=8.7$, p=0.034. For MeO-S-DS is1, degree of freedom=3; n=3-4 per genotype, $\chi^2=11.83$, p=0.008.

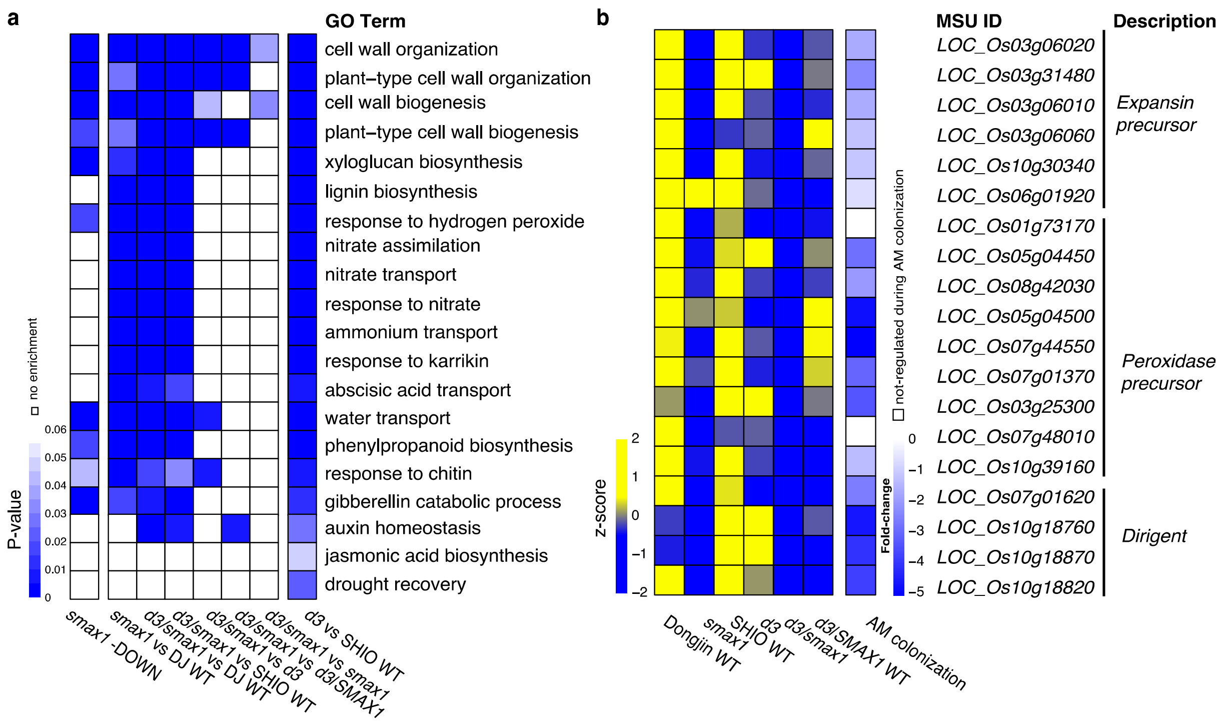

Supplementary Figure 19. Gene ontology enrichment analysis of genes down-regulated by smax1 mutation.

a. Gene Ontology (GO) enrichment analysis of the *smax1*-DOWN gene list. The gene enrichment rate is shown in *p*-value scale (<0.05), where lower values indicate a more significant enrichment. **b.** Relative gene expression of *smax1*-DOWN genes belonging to the GO-term 'cell wall organisation' was visualised relative to mean median-value (bar, z-score 0). The decreased gene expression after AM colonization is shown in fold-change (FDR adjusted p<0.1) relative to non-colonised crown roots [1].

Supplementary Table 1. Mapping summary of the RNAseq reads

Sample description	Sample name	Total sequenced reads	Cleaned reads	Concordantly mapped reads	Discordantly mapped reads	Mapping rate (%)
Dongjin_wild-type_replicate 1	dj_m1	10,030,038	4,493,252	4,279,232	14,563	95.56
Dongjin_wild-type_replicate 2	dj_m2	11,498,971	5,757,605	5,591,957	29,557	97.64
Dongjin_wild-type_replicate 3	dj_m3	13,207,892	6,746,622	6,422,424	22,905	95.53
*smax1*_replicate 1	*smax*_m1	14,025,502	7,447,465	6,896,779	16,887	92.83
*smax1*_replicate 2	*smax*_m2	11,663,778	5,306,492	5,040,876	16,027	95.30
*smax1*_replicate 3	*smax*_m3	9,668,089	5,336,818	5,023,788	18,446	94.48
Shiokari_wild-type_replicate 1	shio_m1	21,605,044	9,991,527	9,579,706	30,887	96.19
Shiokari_wild-type_replicate 2	shio_m2	10,791,912	4,380,601	4,124,420	16,475	94.53
Shiokari_wild-type_replicate 3	shio_m3	12,876,835	6,103,478	5,900,692	13,896	96.91
*d3*_replicate 1	*d3*_m1	19,622,513	9,599,364	9,137,842	42,882	95.64
*d3*_replicate 2	*d3*_m2	18,341,606	6,770,974	6,588,801	25,358	97.68
*d3*_replicate 3	*d3*_m3	19,629,432	8,728,850	8,444,123	30,320	97.09
*d3/smax1*_replicate 1	*dko*_m1	16,032,279	8,481,907	7,911,392	30,319	93.63
*d3/smax1*_replicate 2	*dko*_m2	18,236,907	7,610,722	7,317,523	32,520	96.57
*d3/smax1*_replicate 3	*dko*_m3	13,422,378	6,688,633	6,334,947	25,281	95.09
*d3_SMAX1*_replicate 1	*d3smax*_m1	14,066,204	6,454,430	6,300,901	25,424	98.02
*d3_SMAX1*_replicate 2	*d3smax*_m2	13,333,178	4,808,074	4,643,667	14,494	96.88
*d3_SMAX1*_replicate 3	*d3smax*_m3	35,593,110	18,799,653	18,322,273	77,449	97.87

Supplementary Table 2. Functionally characterised *smax1*-UP genes in AM symbiosis

Gene name	MSU ID	Description	*smax1*-UP[a]	AM conserved[b]	AM induced[c]	AM phenotype	Reference
CCD7	*LOC_Os04g46470*	carotenoid cleavage dioxygenase 7	I		Yes	Reduced colonization	2[d]
CCD8	*LOC_Os01g54270*	carotenoid cleaving deoxygenase 8	I		Yes	Reduced colonization	2[d]
Exo70	*LOC_Os11g01050*	exo70 exocyst complex subunit domain containing protein	I		Yes	Stunted arbuscule	3
AMT3;1	*LOC_Os01g65000*	ammonidum transporter	I		Yes	Suppression of Mtpt4	4
SYMRK	*LOC_Os07g38070*	LRR receptor-like kinase	II	Yes		Infection failure	5
CYCLOPS	*LOC_Os06g02520*	Transcription factor	III	Yes	Yes	Infection failure	6[d]
NFR5	*LOC_Os03g13080*	LysM receptor kinase	III	Yes	Yes	Reduced colonization or AM marker gene experession	7,8[d]
ZAS	*LOC_Os09g15240*	Zaxinone synthase	III	Yes	Yes	Reduced colonization	9
PT11	*LOC_Os01g46860*	Phosphate transporter	III	Yes	Yes	Stunted arbuscule	10[d]
CCaMK	*LOC_Os05g41090*	Calcium/calmodulin-dependent protein kinase	IV			infection failure	6[d]
DXS2	*LOC_Os07g09190*	1-deoxy-D-xylulose 5-phosphate synthase 2	IV			Stunted arbuscule	11
NSP2	*LOC_Os03g15680*	GRAS transcription factor	IV			Reduced colonization	12

[a] Group corresponds to Fig.4a.Group I; induced in *smax1* and by AM symbiosis, Group II; AM conserved genes induced in smax1, Group III; AM conserved genes induced in *smax1* and by AM symbiosis, Group IV; induced in *smax1*

[b] AM conserved genes [13].

[c] Rice crown roots, fold-change (mock vs inoculated), FDR P<0.1 [1]

[d] References characterized in rice were provided only.

Supplementary Table 3. Primers used in this study

Purpose	Gene	MSU ID	Primer name	Pimer sequence	Reference
reference for *smax1* allele test RT- PCR	*Cyclophilin2 (CP2)*	*LOC_Os 02g02890*	CP2 RT-F	TCCCAGTTCTTCATCTGCAC	This study
			CP2 RT-R	ACCAAACCATGGGCGATCT	
reference for *smax1* allele test qRT- PCR			CP2 qRT-F	GTGGTGTTAGTCTTTTTATGAGTT CGT	Gutjahr et al., 2008 (ref. 6)
			CP2 qRT-R	ACCAAACCATGGGCGATCT	
referencefor cDNA synthesis of 5'UTR of *SMAX1* (*CP2* is amplified as a control)			CP2 RT-R	ACCAAACCATGGGCGATCT	This study
			SMAX1-13R	TATGGTGCTAAGATCCGCCCT	
positive control for genotyping			CP2 RT-F	TCCCAGTTCTTCATCTGCAC	This study
			Cyp2-859R	GCGATATCATAGAAGCAGCGAC	
SMAX1 terminator	*SMAX1*	*LOC_Os 08g15230*	3H6	cactctgtgaagaccagcttAGAGGGAACT GGGGGGAGATAAAT	This study
			3H8	cacttcgtagaagactgagcgaagctgtgagaa accgagcaatgcct	
smax1 allele RT-PCR			A	TGCTTCTTTCGTTTGTTCCAAG	This study
			A'	ATTTTCTACCTTTGCAAACTCTAAT	
			B	CCCAAGCGGGCAAATGTAA	
			B'	AAATGCGAATAGAATCTTCGTCT	
			C	TGCTATCACCTGCACTTCGG	
			C'	GTGGCGTGAATGTGGCAAG	
			D	TGAATCCCAGTTGGGAGAAG	
			D'	TTACCAAGGCTGACCTCACG	
			E	GCAGAACCCATTCTCGGTGA	
			E'	ATGACCGATTCAAATTTCGC	
			F	AGGCATGGAGGTTATAGATCT	
			F'	ACCGATCAATCCTTGCAA	
qRT-PCR	*Ri EF1a*		Ri EF1a F	GCTATTTTGATCATTGCCGCC	Perez Tienda et al., 2014 (ref. 14)
			Ri EF1a R	TCATTAAAACGTTCTTCCGACC	
	GAPDH	*LOC_Os 08g03290*	qOs GAPDH F1	CTGATGATATGGACCTGAGTCTA CTTTT	Gutjahr et al., 2008 (ref. 6)
			qOs GAPDH R1	CAACTGCACTGGACGGCTTA	
	Polyubiquitin	*LOC_Os 06g46770*	qOsUbiQ F1	CATGGAGCTGCTGCTGTTCTAG	Gutjahr et al., 2008 (ref. 6)
			qOsUbiQ R1	CAGACAACCATAGCTCCATTGG	
	AM1	*LOC_Os 04g04750*	qAM1F	ACCTCGCCAAAATATATGTATGCT ATT	Gutjahr et al., 2008 (ref. 6)
			qAM1R	TTTGCTTGCCACACGTTTTAA	
	AM3	*LOC_Os 01g57400*	qAM3 F	CTGTTGTTACATCTACGAATAAGG AGAAG	Gutjahr et al., 2008 (ref. 6)
			qAM3 R	CAACTCTGGCCGGCAAGT	
	PT11	*LOC_Os 01g46860*	qPT11 F	GAGAAGTTCCCTGCTTCAAGCA	Gutjahr et al., 2008 (ref. 6)
			qPT11 R	CATATCCCAGATGAGCGTATCAT	

	AM14	*LOC_Os 11g26140*	qAM14 CG F	CCAACACCGTTGCAAGTACAATA C	Gutjahr et al., 2008 (ref. 6)
			qAM14 CG R	GCACTTTGAAATTGGACTGTAAGA AA	
	SMAX1	*LOC_Os 08g15230*	q3E6-SMAX1 3F	AGGCATGGAGGTTATAGATCT	This study
			q3E7-SAMX1 4R	ACCGATCAATCCTTGCAACA	
	D3	*LOC_Os 06g06050*	qD3 F1	TTGAGGTGCAACTGAACAGC	
			qD3 R1	TGGCACCATCCAGATAAATC	
	DLK2a	*LOC_Os 05g15240*	qDLK2a F1	CGATGTTGCCATATAGGTTGTGC	
			qDLK2a R1	ACAAGGGAGCACACATGCAG	
	NFR5	*LOC_Os 03g13080*	qNFR5 F1	ACGCGTTCGAGAGGCTATG	
			qNFR5 R1	TATCTAGCTGCCACCTCGTTC	
	LYK1	*LOC_Os 01g36550*	qLYK1 F1	GCCCTGAAATGAGGGAGGTT	
			qLYK1 R1	ACCATTGGAAACGCCACTGA	
	KIN6	*LOC_Os 04g39180*	qKIN6 F1	GGATACGTCGATCCAGAGT	
			qKIN6 R1	CATGGAAGCCATTCGATCC	
Genotyping *d14l-1, d14l-2*	*D14L*	*LOC_Os 03g32270*	g91gn F1*	TGAAGCCATGGGGTCAAACT	This study
			g91gn R1	CCCTCAGACGGCATTACCTC	
Genotyping *d14l-3*			g98 gnF1	CCTTTTCTGGTCCCGTTCATTC	
			g98 gnR1*	GCTTCGCACATCACTCTGGA	
qRT-PCR of *d14l* mutants			qD14L F1	GAAGCCATGGGGTCAAACTA	Gutjahr et al., 2015 (ref. 1)
			qD14L R1	GCGGCTAAACTCCTGAACTG	
			qD14L F2	TCCCTGTCTTGCTTCGACAC	
			qD14L R2	AGCTCTAGGCGGAATGGTTG	
Genotyping	*CAS9*		CAS9 F1	CGATCAGCTTGTCGGAGTTG	
			CAS9 R1	GACGTGGACCATATTGTGCC	
Checking gDNA contamination after cDNA synthesis	*GAPDH*	*LOC_Os 08g03290*	2E4 GAPDH F1	AGGTTCTTCCTGATTTGAATGG	
			qOs GAPDH CG R1	CAACTGCACTGGACGGCTTA	
Genotyping for Hygromycin	*HPT*		HygF-UP	gtttatcggcactttgcatcggccg	
			HygR-UP	gatttgtgtacgcccgacagtcc	

References

1. Gutjahr C*, et al.* Transcriptome diversity among rice root types during asymbiosis and interaction with arbuscular mycorrhizal fungi. *Proc Natl Acad Sci U S A* **112**, 6754-6759 (2015).

2. Kobae Y*, et al.* Strigolactone Biosynthesis Genes of Rice are Required for the Punctual Entry of Arbuscular Mycorrhizal Fungi into the Roots. *Plant and Cell Physiology*, pcy001-pcy001 (2018).

3. Zhang X, Pumplin N, Ivanov S, Harrison MJ. EXO70I Is Required for Development of a Sub-domain of the Periarbuscular Membrane during Arbuscular Mycorrhizal Symbiosis. *Curr Biol* **25**, 2189-2195 (2015).

4. Breuillin-Sessoms F*, et al.* Suppression of Arbuscule Degeneration in Medicago truncatula phosphate transporter4 Mutants is Dependent on the Ammonium Transporter 2 Family Protein AMT2;3. *Plant Cell* **27**, 1352-1366 (2015).

5. Endre G, Kereszt A, Kevei Z, Mihacea S, Kaló P, Kiss GB. A receptor kinase gene regulating symbiotic nodule development. *Nature* **417**, 962 (2002).

6. Gutjahr C*, et al.* Arbuscular mycorrhiza-specific signaling in rice transcends the common symbiosis signaling pathway. *Plant Cell* **20**, 2989-3005 (2008).

7. Miyata K*, et al.* Evaluation of the Role of the LysM Receptor-Like Kinase, OsNFR5/OsRLK2 for AM Symbiosis in Rice. *Plant Cell Physiol* **57**, 2283-2290 (2016).

8. He J*, et al.* A LysM Receptor Heteromer Mediates Perception of Arbuscular Mycorrhizal Symbiotic Signal in Rice. *Mol Plant* **12**, 1561-1576 (2019).

9. Wang JY*, et al.* The apocarotenoid metabolite zaxinone regulates growth and strigolactone biosynthesis in rice. *Nat Commun* **10**, 810 (2019).

10. Yang SY*, et al.* Nonredundant regulation of rice arbuscular mycorrhizal symbiosis by two members of the phosphate transporter1 gene family. *Plant Cell* **24**, 4236-4251 (2012).

11. Floss DS, Hause B, Lange PR, Kuster H, Strack D, Walter MH. Knock-down of the MEP pathway isogene 1-deoxy-D-xylulose 5-phosphate synthase 2 inhibits formation of arbuscular mycorrhiza-induced apocarotenoids, and abolishes normal expression of mycorrhiza-specific plant marker genes. *Plant J* **56**, 86-100 (2008).

12. Liu W*, et al.* Strigolactone biosynthesis in Medicago truncatula and rice requires the symbiotic GRAS-type transcription factors NSP1 and NSP2. *Plant Cell* **23**, 3853-3865 (2011).

13. Bravo A, York T, Pumplin N, Mueller LA, Harrison MJ. Genes conserved for arbuscular mycorrhizal symbiosis identified through phylogenomics. *Nat Plants* **2**, 15208 (2016).

14. Perez-Tienda J, Correa A, Azcon-Aguilar C, Ferrol N. Transcriptional regulation of host NH4 transporters and GS/GOGAT pathway in arbuscular mycorrhizal rice roots. *Plant Physiol Biochem* ***75***, 1-8(2014).
