# Supplementary material for: The negative regulator SMAX1 controls mycorrhizal symbiosis and strigolactone biosynthesis in rice
Source: Nat Commun. 2020 Apr 30;11:2114. doi: 10.1038/s41467-020-16021-1 (PMC7193599; doi:10.1038/s41467-020-16021-1)
Supplement: Supplementary file 3 — Description of Additional Supplementary Files [file 41467_2020_16021_MOESM3_ESM.docx]

**Description of Additional Supplementary Files**

**File name:** Supplementary Data 1

**Description:** Protein sequences used for phylogenetic tree of SMAX1

**File name:** Supplementary Data 2

**Description:** FPKM of all genes

**File name:** Supplementary Data 3

**Description:** FPKM of genes that are differentially expressed at least one pair-wise comparison

**File name:** Supplementary Data 4

**Description:** GO term analysis for up-regulated genes of all pairwise comparisons

**File name:** Supplementary Data 5

**Description:** List of smax1-UP

**File name:** Supplementary Data 6

**Description:** GO term analysis of smax1-UP

**File name:** Supplementary Data 7

**Description:** List of smax1-DOWN

**File name:** Supplementary Data 8

**Description:** GO enrichment analysis for down-regulated genes of all pairwise comparisons

**File name:** Supplementary Data 9

**Description:** GO term analysis of smax1-DOWN

**File name:** Supplementary Data 10

**Description:** AM conserved genes in rice

**File name:** Supplementary Data 11

**Description:** List of genes required smax1mutation for gene expression
